# Prof. Sandford Eastman

**Published:** 1874-01

**Authors:** 


### Obituary.

It becomes our painful duty to announce through the columns of this Journal, the death on January 8th, at Riverside, California, of Prof. SANDFORD EASTMAN, M. D., formerly of this city. Dr. EASTMAN was born at Lodi, Seneca County, N. Y., Sept. 29th, 1821. His father was a physician who recognized the value of educational advantages, and gave his son the advantages of a thorough education. At the age of fifteen he entered Amherst College, graduating four years later. After leaving College, he taught school for a few years. Having persued to some extent the study of medicine under his father's direction, he resolved to adopt it as a profession, and in 1849 he came to Buffalo, where he entered the office of Prof. Jas. P. White, as a student. After attending three courses of lectures he graduated at the Buffalo Medical College in 1851. Commencing without the aid of influential friends, he soon gained a position of honor among his professional brethren, and won the esteem and confidence of the community. In 1859 he was appointed Professor of Anatomy in the Medical Department of the University of Buffalo, which position he held until 1870, when ill health compelled him to resign the position. On the acceptance of his resignation he was made Emeritus Professor of Anatomy and Clinical Surgery. As a teacher, Prof Eastman was beloved and respected by his pupils, and the news of his death will be received with a feeling of deep sorrow by those who have been priviledged to listen to his teachings.

Prof. Eastman undoubtedly adds another to the long list of those whose lives have fallen a sacrifice to the dangers which constantly beset the path of the medical man and more especially those engaged in a Surgical practice. While making an amputation at the Hospital of the Sisters of Charity for disease of the knee-joint some of the virus was absorbed into his system through an abrasion on his thumb, pyæmia resulted and a abscess formed in one of his lungs already weakened. This condition did not however, deter him from practicing his profession as vigorously as his weakened system would allow, and when remonstrated with for giving so much of his time to patients who could make him no return, when his health scarcely allowed him to attend even to those who could make him some pecuniary return, he replied that he must attend to his poor patients for they depended upon him.

Finding that he could not stand our severe winters he removed to California and for a time seemed to improve. Early in the fall he undertook an excursion to the mountains by which his health, which had declined somewhat, was improved. But here we again see his entire forgetfulness of self when what seemed to him duty's call summoned him elsewhere, while enjoying the change which the mountain air produced he was summoned to attend a friend at Riverside. Although his friends and relatives attempted to dissuade him from returning, he went back and nursed his friend through a long and severe illness, from which he recovered. Almost immediately upon the recovery of his patient Prof. Eastman had an attack of hæmoptysis from which he never recovered.

Prof. Eastman was one of the Surgeons to the Buffalo General Hospital and to the Hospital of the Sisters of Charity. He was an active member of the Buffalo Medical Association and of the Erie County Medical Society, and did much to increase the usefulness of these organizations. He was also a permanent member of the New York State Medical Society. For several years he filled the office of Health physician, giving to its duties all of his accustomed vigor and zeal.

Taken away in the prime of life we shall long mourn his departure. Beloved by all both in professional and social circles, we shall search long before finding one to fill his place. To those who had the pleasure of his friendship and acquaintance his death will cause a loss which can never be replaced. He was a true type of the noble self-sacrificing christian physician.

At a special meeting of the Buffalo Medical Association January 10th, feeling tributes were paid to his memory by Drs. White, Rochester and Miner, and the following resolutions were drawn up by a committee consisting of Drs. White, Rochester, Samo and Miner:

*Resolved*, That, in the death of Dr. Eastman, the Buffalo Medical Association mourns the loss of one who stood among its most honored members; one who largely contributed to its reputation, both at home and abroad; and who to many of us stood in the endeared relation of instructor in the first principles of our profession; and though years have passed since he met with us, the recollection of his services as a member, of his genial qualities as a companion, and of his character as a gentleman, will long be cherished by this Association.

*Resolved*, That while the death of such a man makes a gap in the professional ranks that can not easily be filled, we feel that his influence and example are not lost; we point to him with pride and are thankful that he lived.

*Resolved*, That to those more intimately related to him, upon whom his loss must fall most crushingly, to his bereaved family, we tender our deepest sympathies.

*Resolved*, That a copy of the foregoing be sent to the family; a copy also furnished the Buffalo Medical Journal and the daily papers for publication, and a copy preserved in the records of this Association.

At the regular meeting of the Erie County Med cal Society held January 13th, the following Resolutions were adopted:

Your Committee, to whom was given the painful task of putting into form the expression of this Society with reference to the death of Prof. Sandford Eastman, beg to say that,

*Whereas*, It is but too common to find the great and the good everywhere removed from that sphere of usefulness for which Providence and natural capacity has wonderfully fitted them; and

*Whereas*, It has pleased God to remove from among us, in the person of Sandford Eastman, late Professor of Anatomy in the University of Buffalo, one who was in an eminent degree possessed of those qualifications that rendered his life so useful, and in consequence, its prolongation so much to be desired; but

*Whereas*, It has otherwise been ordered by Him who rules all things according to His will; be it therefore

*Resolved*, That we deeply sympathize with his bereaved family in their irreparable loss, and offer them our heartfelt condolence.

*Resolved*, That this expression of our feeling be published in the papers, and a copy sent to his family.

JOHN CRONYN,

T. M. JOHNSON, } COMMITTEE.

WM. RING,

---

At a Special Meeting of the Faculty of the Buffalo Medical College, upon the announcement of the death of Prof. Sandford Eastman, a committee was appointed, consisting of Profs. James P. White, T. F. Rochester and M. G. Potter, to present resolutions expressive of the loss sustained in his death.

The following was unanimously adopted:

*Whereas*, It has pleased an All-wise Providence to remove from earth our friend and former colleague, Prof. Sandford Eastman. Therefore,

*Resolved*, That while we bow submissively to the will of God, we sadly deplore the loss of one whose unfaltering energy and enthusiastic zeal won our highest admiration, making him at once a wise co-operator in medical teaching and emphatically the students' friend.

*Resolved*, That while we mourn the circumstance of failing health which compelled the "Lamp of Life" to fade in a distant state, and deprived us of the sad privilege of ministering to our brother in his last days of earth, we will emulate his pure example and all the virtues which cluster so tenderly about the christian teacher, physician and friend.

*Resolved*, That we tender to the mourning family our heartfelt sympathy in their bereavement, and commend them to the loving kindness and tender mercy of the widow's and the orphan's God.

